# Detrimental to public health: Royal Australasian College of Physicians’ recent policy on infant circumcision

**DOI:** 10.1038/s41390-024-03190-8

**Published:** 2024-04-13

**Authors:** Brian J. Morris, Jeffrey D. Klausner

**Affiliations:** 1 School of Medical Sciences, Building F13, University of Sydney, NSW, Australia; 2 Department of Medicine, Population and Public Health Sciences, Keck School of Medicine of the University of Southern California, Los Angeles, CA USA

The RACP’s latest infant male circumcision (IMC) policy released in Dec 2022 concludes that it “*believes that the frequency of diseases modifiable by circumcision, the level of protection offered by circumcision and the complication rates of circumcision do not warrant routine infant circumcision in Australia or Aotearoa New Zealand*”.^1^ This long-standing position has led to IMC bans in all public hospitals. In contrast, the American Academy of Pediatrics (AAP) policy in 2012 (expired 2017) states that “*the benefits of circumcision are sufficient to justify access to this procedure for families choosing it and to warrant third-party payment for circumcision of male newborns*”.^2^ But any pediatric surgery other than for urgent medical reasons is never “*routine*” – it requires parental consent. The AAP policy states that “*It is important that clinicians routinely inform parents of the health benefits and risks of male newborn circumcision in an unbiased and accurate manner*”. It is then up to the parents to decide whether to proceed.

The RACP policy expresses concern that the “*complication rate*” of IMC is “*1.5%, with a range of 0–16%*”, but cite (their ref. 4) a dated study of rate, not risks, of circumcision in Australia. Those figures actually come from their ref. 3, a 2010 systematic review by Weiss et al. Most studies in Weiss et al. were relatively small and 15 were from developing countries. The 16% figure for the upper limit of the 0–16% range was from a study in Pakistan. The RACP policy failed to mention the largest, most relevant studies cited by Weiss et al. involving 130,475 and 100,157 IMCs in the UK and US, respectively. These found complication rates of 0.20% and 0.19%, respectively, and were cited by the AAP^2^ and the Centers for Disease Control and Prevention (CDC)^3^ in reviews that formed part of their policy recommendations in 2012 and 2018. The CDC’s policy also cited an even bigger study by CDC researchers of 1.3 million IMCs.^4^ That study found risk of adverse events was 0.4% in neonates, but was 20- and 10-fold higher in males aged 1–9 years and ≥10 years, respectively.^4^ Although the CDC study was cited in the RACP policy as ref. 9, its key findings were ignored, consistent with obfuscation.

The RACP policy mentions that the AAP found “*health benefits of infant male circumcision outweigh the risks*”. The CDC policy (not cited by the RACP) went further by stating that benefits exceed risks by “*100:1*”.^3^ The RACP also ignored the “*Evidence-based circumcision policy for Australia*”, a systematic review by the Circumcision Academy of Australia, which, unlike the RACP’s policy statement posted on the internet, was published in a peer-reviewed journal, and listed on PubMed in May 2022.^5^ This contained a risk-benefit analysis for Australia that found “*benefits exceeded procedural risks, which are predominantly minor, by approximately 200 to 1*”, and that “*more than 1 in 2 uncircumcised males will experience an adverse foreskin-related medical condition over their lifetime*”. Another study found that “*An increase in early MC in Australia to mid-1950s prevalence of 85% from the current level of 18.75% would avoid 77,000 cases of infections and other adverse medical conditions over the lifetime for each annual birth cohort*”.^6^

The RACP policy states that “*Topical local anaesthesia is also not suitable for management of pain with circumcision*”. But it failed to note that adequate time must be allowed. The AAP recommended application of topical anesthesia “*60 to 80* *min before the procedure*”, by which time pain levels are minimal. Although “*infant* [general] *anaesthesia is high risk*”, the RACP’s statement to “*delay until after 12 months*” would result in a 20-fold higher incidence of procedural complications.^4^ In contrast, neonatal IMC is simple, quick, low cost, low risk, bleeding is minimal, sutures are not needed, healing is fast, and is convenient as the infant sleeps mostly, so does not disrupt feeding or other activities. Cosmetic outcome is generally good, there is no long-term memory of the procedure, and local anesthesia can be used because unlike a more mobile older infant there is little movement. Delay also exposes the infant to a 10-fold higher risk of urinary tract infections (UTI) in early infancy (see below).

The “*Health conditions to consider*” section contains inaccuracies. Curiously, the RACP policy cites an old systematic review of IMC and UTI in 2005, but fails to cite the most recent (2013) systematic review and meta-analysis of risk of UTI in infancy and across the lifespan that was published in *Journal of Urology*, the official journal of the American Urological Association.^7^ This found that 32% of uncircumcised males will experience a UTI in their lifetime compared with 8.8% of circumcised males, with number needed to treat being 4.29. UTI is 10-times lower in circumcised infant males, so contradicting the RACP’s claim that UTIs are only “*4-5 times lower in circumcised males*”. Febrile UTI incidence is highest (8.7%) in infants aged <3 months. The RACP’s ref. 20 in fact points out that UTI accounts for “5*–14% of emergency department visits by children annually*” and “*most febrile infants with UTI show evidence of renal parenchymal involvement*”. The policy states that “*about 1 in 20,000 children with a history of UTI will develop end-stage renal disease*”, but that figure is not supported by ref. 20 cited, nor by any of their other references. While “*Circumcision in infant males has been shown to be more effective in preventing UTIs than antibiotic prophylaxis alone*”, the RACP policy fails to mention the growing prevalence of antibiotic resistance that may one day make UTI untreatable.

The claim that there is no difference in the proportion of circumcised and uncircumcised men in Australia, the US and UK ever reporting sexually transmitted infections (STIs) is misleading. While being circumcised does not protect against sexually transmitted urethritis, being circumcised does protect against multiple other STIs. As just one example, the large British Natsal-2 study found 74% lower human papillomavirus (HPV) of any type and 86% lower high risk HPV prevalence in circumcised men.^8^ A recent systematic review of 32 studies and meta-analyses found circumcised men had 55% significantly decreased odds of HPV prevalence and a 44% increased rate of HPV clearance.^9^ The RACP policy, however, cites (ref. 29) a small 2008 study in New Zealand to falsely claim lower HPV in young uncircumcised men. Their prediction that HPV vaccination will decrease cervical cancer incidence to “*fewer 4 new cases per 100,000 women by 2028*” appears overly optimistic. In public health, rather than one measure, all effective interventions are normally considered, including both IMC and early adolescent HPV vaccination.

The RACP policy does, however, cite systematic reviews and meta-analyses finding protection against syphilis, genital herpes and chancroid, to which could be added high-risk HPV, *Trichomonas vaginalis*, *Mycoplasma genitalium*, genital ulcer disease, and candidiasis.^5,10^ A claim that circumcision does not reduce risk of STIs in men who have sex with men (MSM) is misleading. While being circumcised does not protect MSM who adopt the receptive role during anal intercourse, it does reduce risk of STIs in MSM who are predominantly insertive.^10^ This applies to HIV,^11^ for which circumcision has been calculated to be cost-effective,^12^ and syphilis.^11^

The RACP policy cites low annual incidence of penile cancer, but not the more relevant statistic of lifetime prevalence (~1 in 1000) which indicates that this devastating disease with low 5-year survival is uncommon, but not rare. The policy disputes an association between circumcision and reduced prostate cancer, but selectively cites 3 small dated studies rather than systematic reviews and meta-analyses, all of which have shown an association of male circumcision, especially IMC, with ~10% lower prostate cancer incidence (see review^5^).

The policy refers to Article 3 of the United Nations Convention on the Rights of the Child that “*requires that, in all actions concerning children, the best interests of the child shall be the primary consideration*”. But Article 24 on the “*right of the child to the enjoyment of the highest attainable standard of health*”, and “*right of access to such health care services*”, explains why, given the evidence, discouraging or denying IMC, has been construed as unethical. Since benefits greatly exceed risks, IMC should be supported, as does case-law. The RACP refers to policies in European countries opposing IMC, but fails to state that, like theirs, these are not evidence-based.
